# Supplementary material for: Incidence of Antibiotic Exposure for Suspected and Proven Neonatal Early-Onset Sepsis between 2019 and 2021: A Retrospective, Multicentre Study
Source: Antibiotics (Basel). 2024 Jun 10;13(6):537. doi: 10.3390/antibiotics13060537 (PMC11200437; doi:10.3390/antibiotics13060537)

Supplementary material

Table S1. Characteristics of participating hospitals.

Hospital (n=15)	Level of Facilities[a]	Minimal gestational age at birth	Guideline utilized during study period	Province	Average birth rates/year[c]
**A**	II	32 weeks	NvK-guideline[b]	Gelderland	2985
**B**	II	32 weeks	NvK-guideline	North-Holland	1387
**C**	II	32 weeks	NvK-guideline	Brabant	3399
**D**	II	32 weeks	NvK-guideline	Overijssel	1687
**E**	I	32 weeks	NvK-guideline	North-Holland	2399
**F**	II	32 weeks	NvK-guideline	South-Holland	2184
**G**	II	30 weeks	NvK-guideline	Brabant	3111
**H**	II	32 weeks	NvK-guideline	Brabant	1034
**I**	II	32 weeks	NvK-guideline	South-Holland	4117
**J**	II	32 weeks	NvK-guideline	North-Holland	2186
**K**	II	32 weeks	NvK-guideline, EOS calculator	Utrecht	2235
**L**	I	32 weeks	NvK-guideline	Drenthe	1120
**M**	II	32 weeks	NvK-guideline	South-Holland	3015
**N**	IV	24 weeks	NvK-guideline	Limburg	1143
**O**	IV	24 weeks	NvK-guideline	South-Holland	2494

[a] Level of care as defined by the American Academy of Pediatrics.
[b] Guideline of the Dutch Association of Paediatrics: Prevention and Treatment of Early-onset Neonatal Infections (2017)
[c]Average birth rate calculated by using birth rates of 2019, 2020 and 2021

Table S2. Birth rates, initiation rates, continuation rates, culture-proven EOS cases and number of treated neonates per culture-proven EOS case for all hospitals per year.

	**Birth rates**				**Antibiotic initiation**					**Antibiotic continuation**						**Culture-proven EOS**					**Burden of treatment vs. burden of disease**	
**Hospital**	**2019 (n)**	**2020 (n)**	**2021 (n)**	**Total (n)**	**2019 (n)**	**2020 (n)**	**2021 (n)**	**Total (n)**	**% of hospital births**	**2019 (n)**	**2020 (n)**	**2021 (n)**	**Total (n)**	**% of initiated**	**% of hospital births**	**2019 (n)**	**2020 (n)**	**2021 (n)**	**Total (n)**	**Incidence per 1000 (hospital birth rate)**	**Number initiated per proven EOS case**	**Number continued per proven EOS case**
A	2895	3033	3026	8954	241	234	183	658	7,3	81	63	46	190	28,9	2,1	6	3	0	9	1,0	73	21
B	1451	1231	1479	4161	55	62	62	179	4,3	15	21	29	65	36,3	1,6	1	1	0	2	0,5	90	33
C	3081	3337	3779	10197	195	185	218	598	5,9	168	171	195	534	89,3	5,2	7	7	6	20	2,0	30	27
D	1636	1643	1781	5060	20	19	36	75	1,5	20	19	16	55	73,3	1,1	3	4	0	7	1,4	11	8
E	2290	2277	2631	7198	39	25	49	113	1,6	12	8	14	34	30,1	0,5	1	1	1	3	0,4	38	11
F	2171	2103	2279	6553	89	96	82	267	4,1	25	15	7	47	17,6	0,7	1	1	2	4	0,6	67	12
G	3071	3037	3226	9334	69	67	97	233	2,5	59	62	83	204	87,6	2,2	4	3	3	10	1,1	23	20
H	1019	1012	1072	3103	6	13	11	30	1,0	3	7	6	16	53,3	0,5	0	1	0	1	0,3	30	16
I	4125	4073	4152	12350	173	208	238	619	5,0	130	147	170	447	72,2	3,6	3	3	2	8	0,6	77	56
J	2131	2106	2322	6559	86	76	85	247	3,8	73	50	60	183	74,1	2,8	2	5	2	9	1,4	27	20
K	2278	2085	2341	6704	78	60	80	218	3,3	66	45	51	162	74,3	2,4	1	3	0	4	0,6	55	41
L	1014	1068	1279	3361	33	31	38	102	3,0	21	20	24	65	63,7	1,9	0	2	0	2	0,6	51	33
M	2964	2935	3147	9046	61	70	45	176	1,9	28	28	18	74	42,0	0,8	5	2	1	8	0,9	22	9
N	1142	1124	1163	3429	158	151	167	476	13,9	79	69	70	218	45,8	6,4	1	4	1	6	1,7	79	36
O	2575	2543	2365	7483	228	273	263	764	10,2	33	41	31	105	13,7	1,4	11	5	8	24	3,2	32	4
**Total**	**33843**	**33607**	**36042**	**103492**	**1531**	**1570**	**1654**	**4755**	**4,6**	**813**	**766**	**820**	**2399**	**50,5**	**2,3**	**46**	**45**	**26**	**117**	**1,1**	**41**	**21**

Figure S1.A. Proportion of neonates initiated on antibiotic therapy per hospital per year

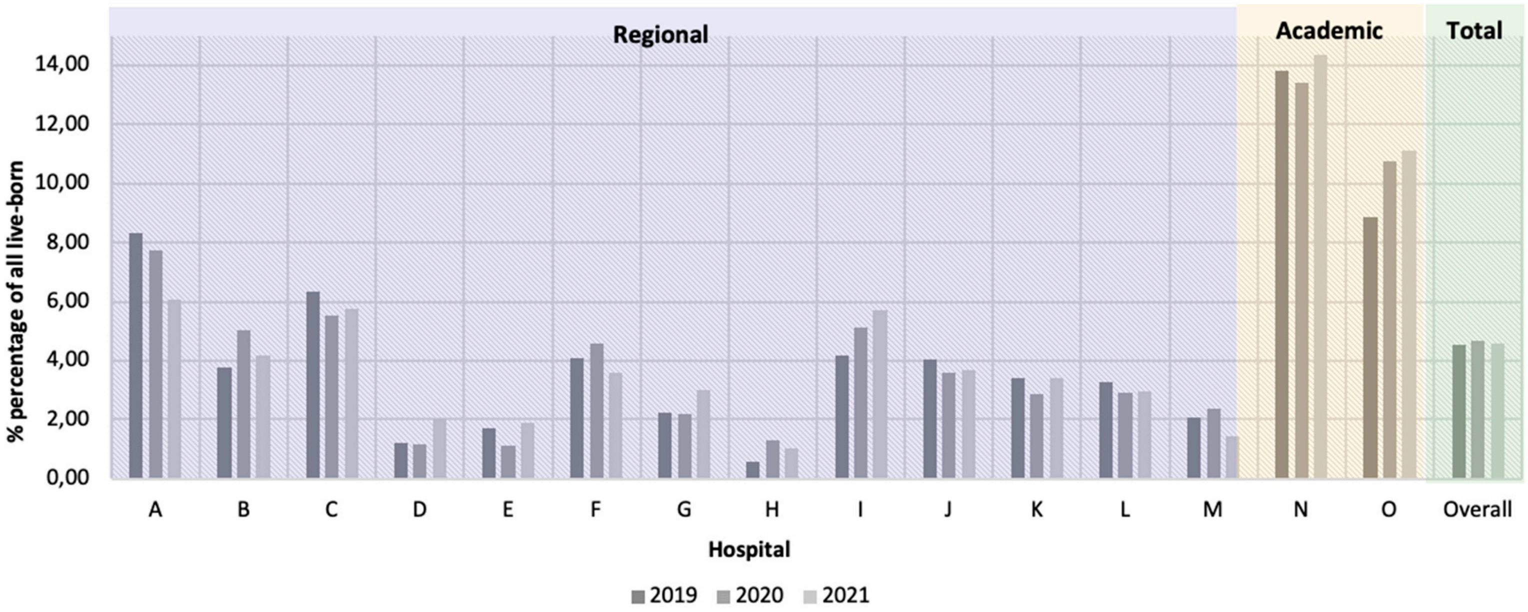

Figure S1.B. Proportion neonates continued on antibiotic therapy for >48 hours per hospital per year

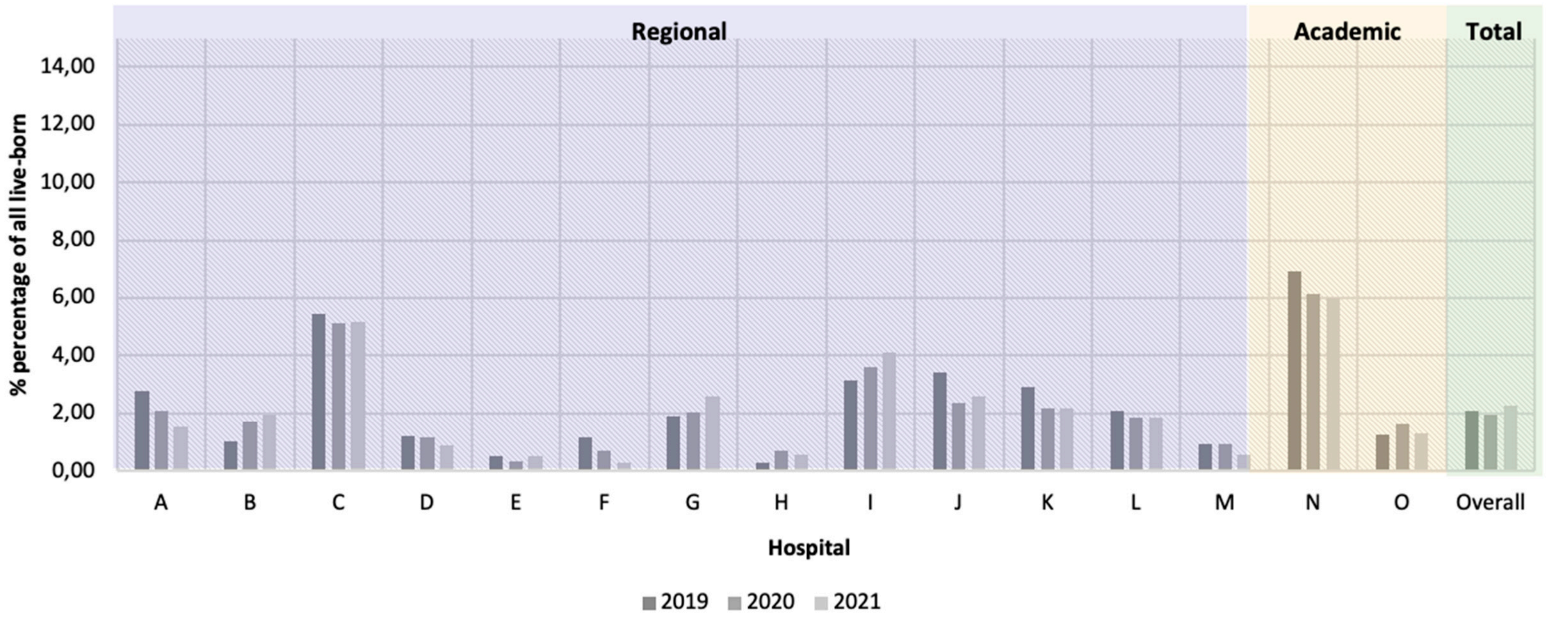

Figure S1.C. Incidence per 1000 live-births per hospital per year

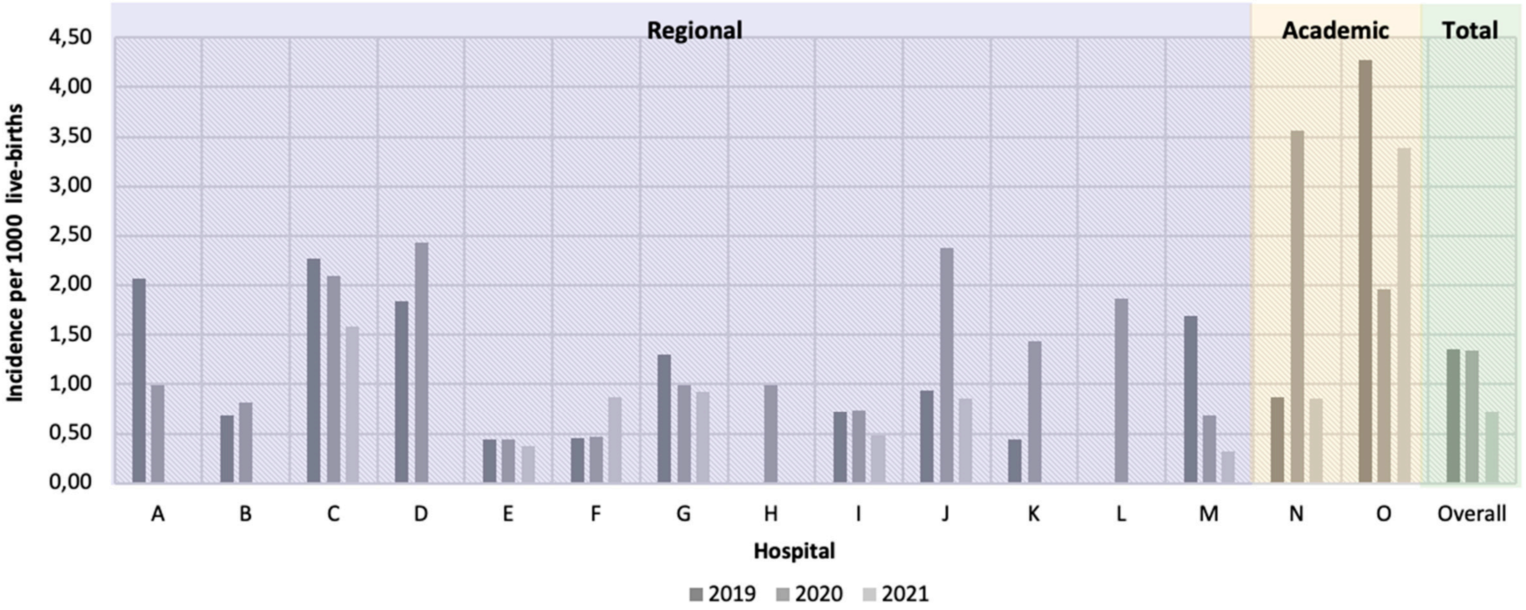

Supplement: Supplementary file 1 [file antibiotics-13-00537-s001.zip › antibiotics-3032875-supplementary.pdf]
